# The Formation of Volume Transmission Gratings in Acrylamide-Based Photopolymers Using Curcumin as a Long-Wavelength Photosensitizer

**DOI:** 10.3390/polym15071782

**Published:** 2023-04-03

**Authors:** Katherine Pacheco, Gabriela Aldea-Nunzi, Agnieszka Pawlicka, Jean-Michel Nunzi

**Affiliations:** 1 Département de Physique, Université d’Angers, 49035 Angers, France; k.b.pacheco.morillo@tue.nl; 2 Department of Chemistry, Queen’s University, Kingston, ON K7L 3N6, Canada; 3 Instituto de Química de São Carlos, Universidade de São Paulo, Av. Trabalhador Sãocarlense 400, São Carlos 13566-590, SP, Brazil; agnieszka@iqsc.usp.br; 4 Department of Physics, Engineering Physics and Astronomy, Queen’s University, Kingston, ON K7L 3N6, Canada

**Keywords:** natural dye, photopolymer, photosensitizer, volume holography

## Abstract

Curcumin, a natural dye found in the *Curcuma longa* rhizome, commonly called turmeric, is used as a photosensitizer in acrylamide-based photopolymers for holographic data storage. We studied the absorbance of photopolymer films that show two absorption bands due to curcumin, acrylamide monomer (AA), and the crosslinking agent N,N′-methylenebisacrylamide (MBA). Analysis of the real-time diffraction efficiency of these films shows a maximum of 16% for the sample with the highest curcumin concentration. Moreover, increasing the curcumin load enhanced the refractive index contrast from 7.8 × 10^−4^ for the photopolymer with the lowest curcumin load to 1.1 × 10^−3^ for the photopolymer with the largest load. The sensitivity and diffraction efficiency of the recorded gratings also increased from 7.0 to 9.8 cm·J^−1^ and from 7.9 to 16% with the increase in curcumin load, respectively. Finally, the influence of NaOH on the photopolymerization of the AA-curcumin-based sample shows a diffraction efficiency increase with the NaOH content, revealing that the curcumin enol form is more efficient as a photosensitizer.

## 1. Introduction

Photopolymers are materials that are photosensitive, so they find modern applications in holography which records and retrieves information by means of the interference and diffraction of light. This property provides a variety of uses, for example, holographic displays and sensors, micro- and nanoelectronics, and 3D and 4D printing [1], for applications in medical materials, printed circuit boards, and microelectromechanical systems (MEMS) [2]. Because of this, photopolymers are widely studied for high-density data storage due to many advantages such as their large refractive index modulation, high optical sensitivity, and low processing cost [3,4]. Photopolymers are made up of one or two co-monomers, a free radical initiator, and a photosensitizer that initiates polymerization by photoinduced charge transfer with the initiator [5]. All these components are dissolved in a polymer matrix that acts as a binder providing the desired thickness to the photosensitive film [4,6]. However, optimization of each component is required to improve the performance of the materials to reach the desired formula. Furthermore, the exposition of this film to light by using an interference pattern induces polymerization and/or crosslinking in the constructive—illuminated—areas, so the monomer polymerizes, which results in concentration gradient formation. Because of this, the new monomers start to migrate from dark to light areas, resulting in local chemical composition variation and a change in density. As a result, there is a change in the refractive index between the illuminated and non-illuminated regions [7].

Turmeric is a yellow-coloured mixture of natural compounds contained in the *Curcuma longa* rhizomes. Turmeric is a typical spice that provides so-called curry powder. The main ingredient in turmeric is curcumin [1,7-bis(4-hydroxy-3-methoxyphenyl)-1,6-heptadiene-3,5-dione], which is a yellow–orange pigment [8]. The compound is present at 1–2 wt% in turmeric [9]. As is well known, curcumin is a colouring agent approved for use in food, drug, and cosmetic products. It is also found as an efficient photoinitiator of polymerization [10]. It has been used as a photolysis sensitizer of diaryliodonium salts to photoinduce the cationic polymerization and copolymerization of styrene and methacrylate [3]. It also acts as an antibacterial agent under visible light illumination [11]. Curcuminoid derivatives are also used as photopolymerization initiators under red and near-infrared light [12].

In this paper, we present curcumin as a photosensitizer of an acrylamide-based photopolymer for the recording of transmission holographic gratings at 488 nm wavelength. Moreover, we studied the effect of dye concentration tuning on the recording wavelength and the angular response of diffraction efficiency (*η*).

## 2. Materials and Methods

Curcumin was isolated from turmeric powder using the method proposed by Anderson et al. [13]. A quantity of 40 g of turmeric powder acquired from a nearby grocery store was added to 100 mL of dichloromethane and stirred at 40 °C for 1 h. The mixture was filtered and concentrated with a rotavapor at 40–50 °C. The oily reddish-yellow residue was stirred overnight with 40 mL of hexane, resulting in a solid. This solid was dissolved in a minimum amount of 99% dichloromethane and 1% methanol solvent mixture and loaded onto a column with 70 g of silica gel. The column was eluted with the same mixture. The least polar-coloured component was concentrated using a rotavapor. The final product was characterized as curcumin by ^1^H NMR with a 125.7 MHz instrument using deuterated DMSO as a solvent. The curcumin was characterized by UV-Vis spectra recorded using a Perkin Elmer Lambda 19 spectrometer.

Pre-polymer solutions were prepared by mixing the acrylamide monomer (AA, Sigma Aldrich Chemie, Saint-Quentin-Fallavier, France), the crosslinking agent N,N′-methylenebisacrylamide (MBA, Fluka, France), the initiator triethanolamine (TEA, Aldrich, France), and the photosensitizer curcumin, at different concentrations in a 13% (*w/v*) poly(vinyl alcohol) aqueous solution (PVA, Sigma Aldrich Chemie, Saint-Quentin-Fallavier, France), in order to obtain a homogenous solution. The compositions of the solutions are given in Table 1. Sample preparation was carried out inside a dark room. The solution was cast on pre-cleaned 2.5 cm × 2.5 cm glass substrates. The samples were left to dry for two days. The thickness of the samples was measured at around 50 μm using a DEKTAK 6M profilometer.

**Figure 1:**
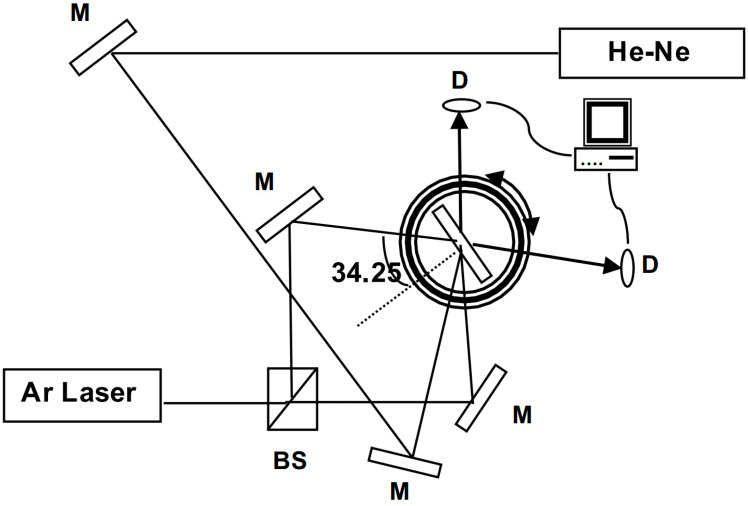
The experimental setup used for recording transmission holographic gratings.

The experimental setup used for recording transmission holographic gratings is illustrated in Figure 1. The gratings were formed from the recombination of two 488 nm beams from an argon (Ar) ion laser with a total intensity of 133 mW·cm^−2^. The angle between the two beams was α = 2 × 34.25°, providing a fringe spacing *λ*/2·sin(α/2) = 0.433 μm, which gives a spatial frequency of 2306 lines/mm. The real-time diffraction efficiency was registered by measuring the diffracted beam from a He-Ne laser beam at 633 nm wavelength at the Bragg angle of 46.8°, using two silicon Hamamatsu photodiodes for reference and signal. This wavelength was chosen to be in a range where the material does not absorb; hence, no polymerization takes place. Diffraction efficiency is defined as the ratio between the diffracted intensity and the sum of the transmitted and diffracted intensities.

## 3. Results and Discussion

**Figure 2:**
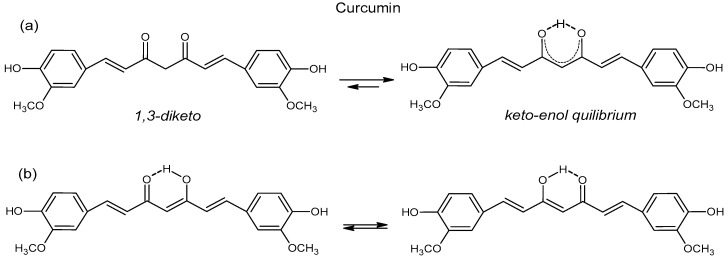
Keto-enol (**a**) and enol-enol (**b**) tautomers of curcumin (adapted from [15]).

The systematic name of curcumin is 1,7-bis(4-hydroxy-3-methoxyphenyl)-1,6-heptadiene-3,5-dione, which means that it is a 1,3-diketone (Figure 2a). There is some confusion in the literature concerning the curcumin form in a solid state. Payton et al.’s [14] curcumin crystal X-ray studies show that it is a keto-enol tautomer, Tewari et al. [15] write that the yellow keto form is predominant in the solid state (Figure 2a), and Akram et al. [16] claim that the enol form is more energetically stable in both a solid state and in a solution. These studies indicate tautomerism in the solid state of curcumin, which corroborates Martin’s work [17], but contradicts Benfenati et al. [18], who claim that tautomers exist only in a solution or a liquid state. In summary, there is still no agreement about keto, enol or keto-enol forms in the solid state of curcumin.

The tautomeric forms of curcumin in solution are more elucidated, and many researchers agree that in neutral, polar, and acidic solutions with pH ≤ 7.4, curcumin is predominant in keto form, and in non-polar and basic solutions, at pH ≥ 8, the enol form occurs [14,15]. However, more recently, Prasad et al. [19] reported on NMR studies of curcumin, demethoxycurcumin, and bisdemethoxycurcumin in solutions, and they found that neutral and acidic solutions favored the keto-enol tautomer and alkaline solutions favored the β-diketone form. Additionally, the enol form has two equivalent tautomers because of intramolecular hydrogen transfer (Figure 2b) [15].

**Figure 3:**
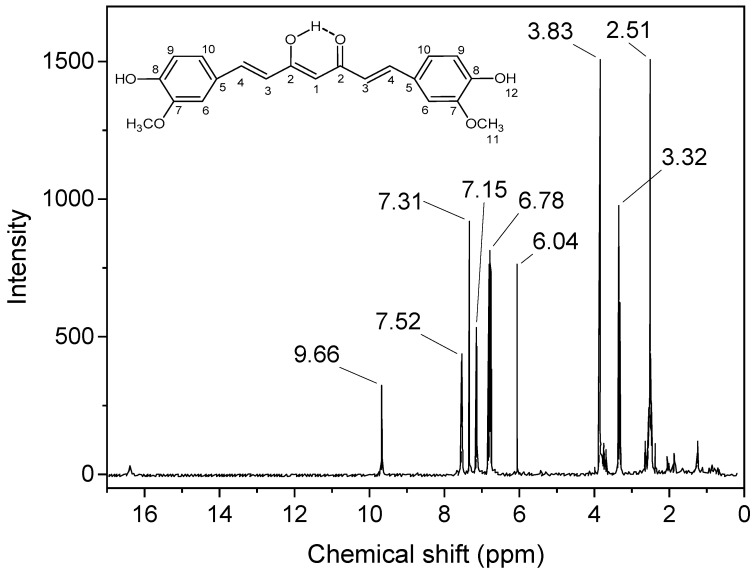
^1^H-NMR of curcumin in DMSO-*d_6_* with curcumin’s hydrogen numbers in the inset.

Aiming to characterize our curcumin, we also performed ^1^H NMR analysis in DMSO-*d_6_* (Figure 3). ^1^H and ^13^C NMR of the extracted and purified curcumin in CDCl_3_ are shown in Figures S1–S4 of the Supplementary Material. Figure 3 reveals a DMSO peak at δ = 2.51 ppm and multiple peaks characteristic of curcumin. The peak at 3.32 ppm is attributed to water in DMSO, and the singlet peak at 3.83 ppm is assigned to 6 hydrogens of both OCH_3_ (H11); at 6.07 ppm to one hydrogen of H1 and 6.78 to H3 [20]; at 7.15 ppm to H10 and 7.31 ppm to H6; 7.52 ppm for hydrogens H4; and at 9.66 ppm to phenolic OH hydrogens (H12) [13,20,21]. Heteronuclear single quantum coherence (HSQC) analysis, which correlates proton–carbon single bonds, confirms our ^1^H and ^13^C NMR peaks’ attributions (Figure S6). Finally, as our ^1^H NMR spectrum results are identical to the ^1^H NMR data reported by Anderson et al. [13], who also analyzed their extracted curcumin sample in DMSO by ^13^C NMR, we conclude that our final product of the turmeric extraction process is curcumin in its hydrogen-bond-stabilized enol form.

**Figure 4:**
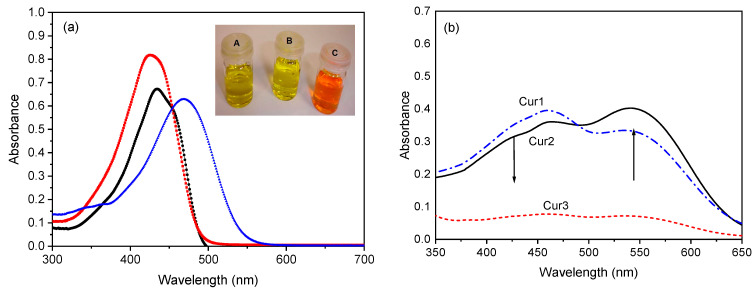
(**a**) Curcumin UV/Vis spectra at 2.7 × 10^−4^ mol·L^−1^ curcumin concentration in anhydrous ethanol (red squares), DMSO (black circles), and aqueous NaOH solution (0.5 mol·L^−1^, blue triangles); (**b**) UV spectra of the cast photopolymers Cur1 (blue dash-dot line), Cur2 (black solid line), and Cur3 (red dash line) as 50 μm-thick films. The inset in (**a**) shows solutions in DMSO (A), ethanol (B), and NaOH (C).

Curcumin solubility differs from organic solvents, and it is insoluble in neutral or acidic water, but it is soluble in a basic NaOH solution. Figure 4a shows the UV-Vis spectra of curcumin solutions in ethanol (Figure 4a red squares), in DMSO (Figure 4a black circles), and in aqueous basic solution (Figure 4a blue triangles). This experiment was performed to show curcumin’s pH sensitivity, which behaves differently in different solvents, mostly because of its phenolic groups [9]. As discussed above, the solubilization of curcumin also results in (or at least increases) the keto-enol tautomerism. Figure 4a shows that all three UV absorption bands are broad, ranging from 300 to almost 500 nm [10]. The absorption maxima occur at 427.3, 433.5, and 469.3 nm for solutions of curcumin in ethanol, DMSO, and NaOH, respectively. These bands are assigned to π-π* curcumin transitions [22] and are at similar wavelengths as observed by others. For example, Crivelo et al. [9] observed that curcumin’s absorption band was 427 nm in a glacial acetic acid solution, which shifted to ~450 nm for the solution in NaOH. It can be seen that as the curcumin solution’s pH changes from acid (ethanol) to basic sodium hydroxide solution (NaOH 0.5 mol·L^−1^), the absorption peak intensity decreases and shifts towards longer wavelengths. This shift of the maximum absorption wavelength of the compound occurs because of the presence of methoxy and hydroxyl groups [9]. The curcumin solution’s UV-Vis spectra bathochromic shift as a function of pH can be observed visually. The ethanol solution colour is pale yellow (Figure 4a inset B), changes to dark yellow for the DMSO solution (Figure 4a inset A), and is reddish-orange for the curcumin-NaOH solution (Figure 4a inset C). Besides the curcumin solutions in C_2_H_5_OH, DMSO, and NaOH 0.5 mol·L^−1^, the UV-Vis spectra were also recorded in CH_2_Cl_2_ and CHCl_3_ (Figure S6). Finally, we also conducted FTIR analyses on the extracted and purified curcumin, and the results are shown in Figures S7 and S8 and are discussed in the Supplementary Material.

Figure 4b shows the absorbance of three cast photosensitive films with different concentrations of curcumin in the UV-Vis range of 350 to 650 nm. Besides curcumin, the samples Cur1, Cur2, and Cur3 contain PVA, acrylamide monomer (AA), the crosslinking agent N,N′-methylenebisacrylamide (MBA), and the initiator triethanolamine (Table 1). Therefore, instead of one absorption band, now there are two bands observed at 460 and 543 nm (Figure 3b) due to π-π* transitions of the keto-enol groups of curcumin [21]. As the concentration of curcumin increases from sample Cur1 to Cur2, the intensity of the band at lower wavelengths decreases and at higher wavelengths, it increases. The Cur3 sample’s band appears to have the same intensity. This change in the UV-Vis spectra can be due either to the polymerization occurring during the analysis or to the interactions between the sample’s constituents.

**Figure 5:**
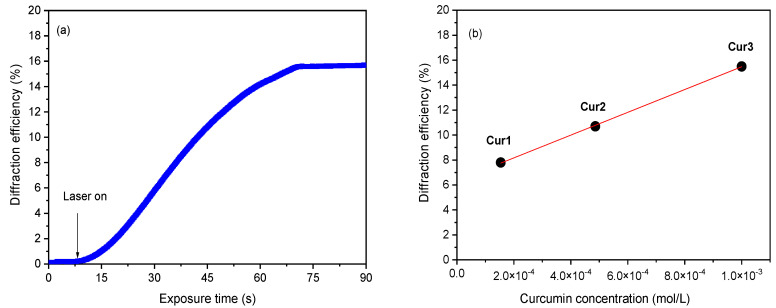
Evolution of the diffraction efficiency (*η*) with the exposure time for Cur3 (**a**) and saturated *η* as a function of curcumin concentration (**b**).

The diffraction efficiency (*η*) of the gratings can be defined by Equation (1) [7].

(1) $$\eta =\frac{I_{D}}{I_{T}+I_{D}}\times 100,$$

where *I_D_* is the diffracted intensity and *I_T_* is the transmitted Ar-ion laser intensity. The exposure time evolution of the diffraction efficiency of the curcumin photopolymer (Cur3) was monitored using the He-Ne laser beam is shown in Figure 5a. There is no induction period after illumination because the diffraction efficiency increases instantaneously and saturates after 60 s of exposure. At 90 s of exposure, it yields up to *η* = 16%.

Figure 5b shows values of the maximum diffraction efficiency as a function of curcumin content in the Cur1, Cur2, and Cur3 samples. It is seen that *η* increases linearly (R^2^ = 0.99) with the curcumin content from 7.8% for Cur1 to 15.5% for Cur3. Previously, it was observed that an increase in the acrylamide monomer promotes an increase of diffraction efficiency in the AA samples with rose bengal dye [7]. In the present study, the same is likely to happen as additional acrylamide monomers may polymerize with larger curcumin concentrations, and this promotes an increase in the diffraction efficiency [23]. Further increases in the curcumin load degraded the quality of the film with non-reproducible properties because of the curcumin’s limited solubility.

The refractive index modulation (Δ*n*), which is the maximum difference between light-exposed and unexposed areas, was calculated from the experimentally measured diffraction efficiency (*η*; Equation (2)) using Kogelnik’s coupled wave theory [24].

(2) $$\eta =\frac{\pi \;\Delta n\;d}{\lambda \;cos\theta_{B}},$$

where *λ* is the reconstruction wavelength, i.e., the wavelength of the probe beam inside the material, θ*_B_* is the Bragg angle inside the material, and *d* is the thickness of the grating. Increasing the curcumin load enhances the refractive index contrast from 7.8 × 10^−4^ for the photopolymer with the lowest curcumin load to 1.1 × 10^−3^ for the photopolymer with the largest load (Table 2).

The sensitivity (*S*) of the photopolymers is defined by Equation (3) [25]:

(3) $$S=\frac{\eta^{1/2}}{I\times \tau \times d}\;,$$

where *η* is the diffraction efficiency, *I* ($I=\frac{2P}{A}\;$, where *P* is the power of each beam and *A* is the illuminated area) is the intensity of light exposure, τ is the exposure time to reach the maximum efficiency, and *d* is the material thickness. Table 2 shows that the increase in curcumin concentration promotes an increase in the sample’s sensitivity from 6.99 to 9.80 cm·J^−1^ for the samples of Cur1 and Cur3, respectively.

**Figure 6:**
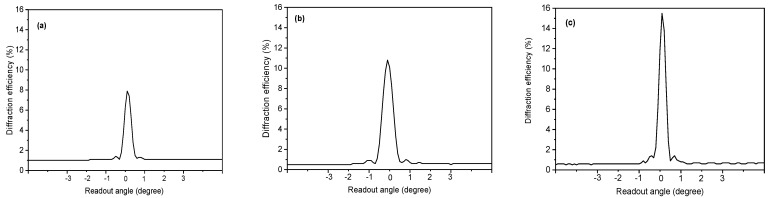
The angular response of the diffraction efficiency for samples Cur1 (**a**), Cur2 (**b**), and Cur3 (**c**).

The angular selectivity of the recorded gratings was investigated by rotating the samples with a resolution of 0.1°. Figure 6 illustrates the angular dependence of the diffraction efficiency at 633 nm with a spatial frequency of 2306 lines/mm. A symmetrical shape around the maximum diffraction efficiency is observed in all photopolymers. By increasing the curcumin concentration, the diffraction efficiency increases from 7.8% for Cur1 to 16% for Cur3, and the full width at half maximum (FHWM) is reduced to 0.5°, showing the selective nature of Bragg diffraction in thick volume holograms [24].

**Figure 7:**
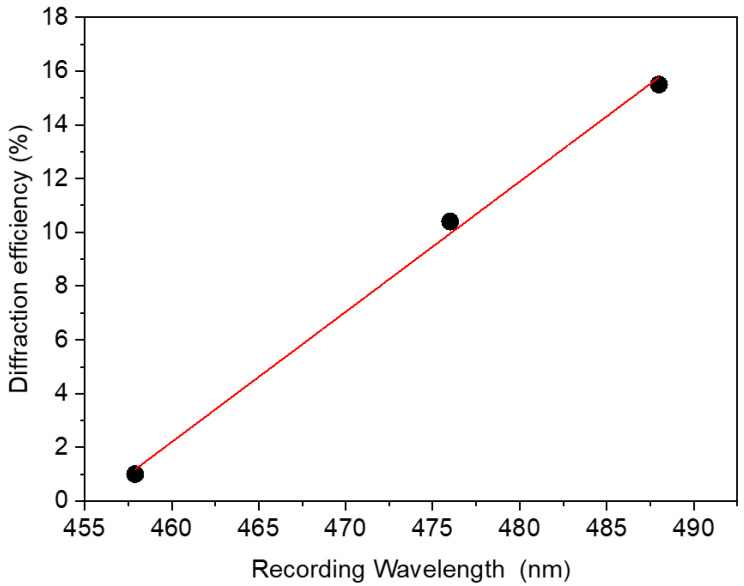
Saturated diffraction efficiency as a function of the recording wavelength with a curcumin concentration of 1.008 × 10^−3^ mol·L^−1^ (Cur3).

The action of the recording wavelength on the Cur3 photopolymer is shown in Figure 7, with different argon-ion laser lines at 488, 476, and 457 nm. The diffraction efficiency increases linearly (R^2^ = 0.99) with the wavelength. This behaviour can be explained as increasing the wavelength increases the fringe spacing, rendering the index contrast larger owing to the photopolymerization process that is limited by the diffusion of the monomers.

Finally, we investigated the influence of the NaOH content in the AA-curcumin-based photopolymers. Therefore, samples with various NaOH content were prepared while keeping the quantity of other reactants identical. Holographic gratings were recorded under identical conditions, i.e., for 90 s exposures at 133 mW·cm^−2^ and 2.15 × 10^−4^ mol·L^−1^ curcumin concentration. Table 3 summarizes the effect of basicity on the diffraction efficiency. The diffraction efficiency increases with the NaOH content, showing that the enol form (Figure 3 inset) is a more efficient photosensitizer. In summary, from the results above it is clear that curcumin is an interesting and natural dye that can be suitable in applications for optical storage through dual-frequency holography [26].

## 4. Conclusions

Curcumin is an efficient photosensitizer for volume data storage applications. It permits the creation of transmission gratings in an acrylamide-based photopolymer. Diffraction efficiency increases noticeably with the curcumin concentration, up to a refractive index modulation of 1.1 × 10^−3^. The enol form favoured in basic media appears as the most efficient photosensitizer. The results show that curcumin is a promising natural dye that can find applications for optical storage through dual-frequency holography.

## Figures and Tables

**Table 1:** The molar concentration of the samples incorporated in the 13% (*wt/v*) PVA solutions.

Sample	AA (mol·L^−1^)	MBA (mol·L^−1^)	TEA (mol·L^−1^)	Curcumin (mol·L^−1^)
**Cur1**	0.45	0.05	0.6	1.54 × 10^−4^
**Cur2**	0.45	0.05	0.6	4.86 × 10^−4^
**Cur3**	0.45	0.05	0.6	1.01 × 10^−3^

**Table 2:** Optical properties of curcumin photopolymers after 60 s exposure.

	*η* (%)	*d* (μm)	Δ *n*	*S* (cm·J^−1^)
**Cur1**	7.8	50	7.8 × 10^−4^	7.0
**Cur2**	11	50	7.98 × 10^−4^	8.2
**Cur3**	16	50	1.11 × 10^−3^	9.8

**Table 3:** Diffraction efficiency (*η*) for different NaOH concentrations (curcumin concentration is 2.18 × 10^−4^ mol·L^−1^).

Basis Content [NaOH] (mol·L^−1^)	*η* (%)
0.290	4.7
0.200	2.9
0.083	1.2

## Data Availability

Data available upon request.

## Supplementary Materials

The following supporting information can be downloaded at: https://www.mdpi.com/article/10.3390/polym15071782/s1, Figure S1. 1H NMR spectrum of extracted curcumin in CDCl3; Figure S2. 13C NMR spectrum of extracted curcumin in CDCl3; Figure S3. 1H NMR spectrum of purified curcumin in CDCl3; Figure S4. 13C NMR spectrum of purified curcumin in CDCl3; Figure S5. Identification of H and C atoms in curcumin molecule used in this work; Figure S6. HSQC whole (a) and amplified (b) spectra of purified curcumin; Figure S7. The UV-Vis spectra of curcumin solutions in CH2Cl2, C2H5OH, DMSO, NaOH 0.5 mol/L, and CHCl3; Figure S8. FTIR spectrum of extracted curcumin; Figure S9. FTIR spectrum of purified curcumin; Table S1. 1H and 13C NMR chemical shifts of protons and carbons in extracted (EC) and purified curcumin (PC); Table S2. Summary of CH2Cl2, C2H5OH, DMSO, NaOH, and CHCl3 relative polarities, polarity indexes, type of solvent, pH and curcumin peaks in these solvents [19,27,28].
