# Supplementary material for: The Formation of Volume Transmission Gratings in Acrylamide-Based Photopolymers Using Curcumin as a Long-Wavelength Photosensitizer
Source: Polymers (Basel). 2023 Apr 3;15(7):1782. doi: 10.3390/polym15071782 (PMC10096970; doi:10.3390/polym15071782)
Supplement: Supplementary file 1 [file polymers-15-01782-s001.zip › polymers-2022793-supplementary.pdf]

Supplementary Material

Formation of Volume Transmission Gratings in Acrylamide-based Photopolymers using Curcumin as a Long-wavelength Photosensitizer

Katherine Pacheco, Gabriela Aldea-Nunzi, Agnieszka Pawlicka and Jean-Michel Nunzi

The curcumin was extracted from commercial turmeric powder and purified, using chromatography 20 mm/300 mm column filled with 20 g of silica gel 60, and CH_2Cl_2 99% and MeOH 1% solvent.

NMR results

The ^1H and ^{13}C NMR spectra of extracted and purified curcumin were obtained with NMR 500/54 Premium Shielded, Agilent Technologies. Figure S1 shows the ^1H NMR spectrum of extracted curcumin in $CDCl_3$.

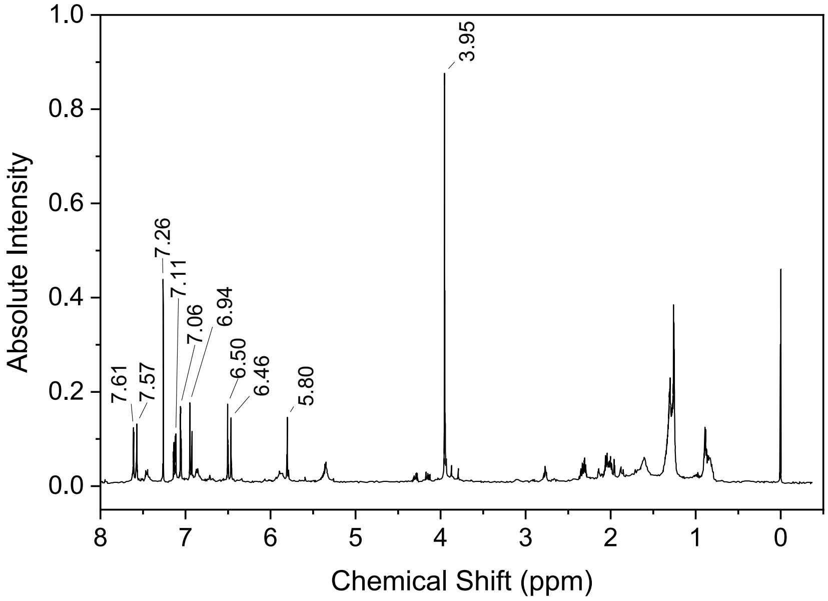

Figure S1. ^1H NMR spectrum of extracted curcumin in $CDCl_3$.

Analyzing the ^1H NMR results in Fig. S1 it seems that curcumin extracted from turmeric and not purified exists in keto-enol form. This is evidenced by chemical shifts in 6.46-6.50 ppm attributed to protons in positions 3 and 3′ [1], and there are no peaks around 6.7 ppm, usually

assigned to the β-diketone curcumin form. The non-identified peaks can be assigned to the impurity, probably grease used on the glass joints of the roto evaporator.

Figure S2 shows the ^{13}C NMR spectrum of extracted curcumin.

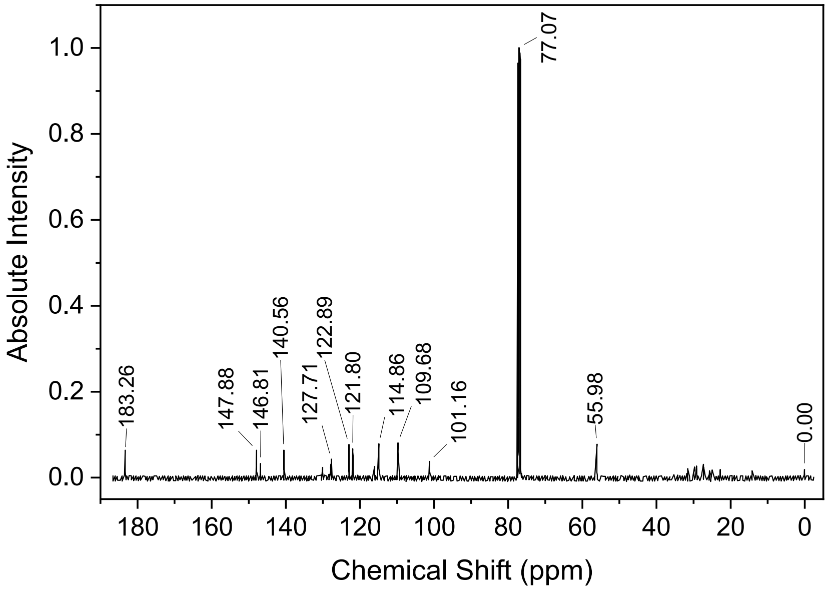

Figure S2. ^{13}C NMR spectrum of extracted curcumin in $CDCl_3$.

Moreover, we also analyzed purified curcumin by ^1H and ^{13}C NMR in $CDCl_3$, and the spectra are shown in Figures S3 and S4.

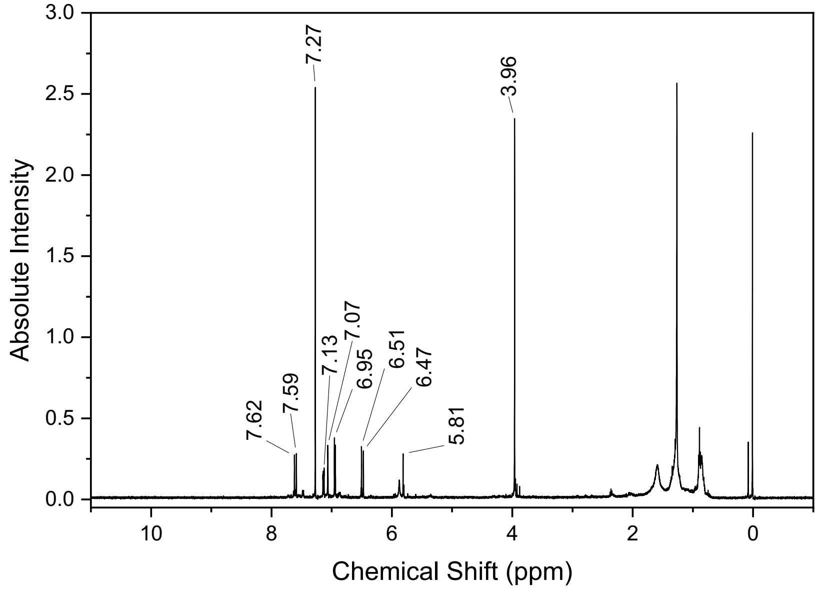

Figure S3. ^1H NMR spectrum of purified curcumin in $CDCl_3$.

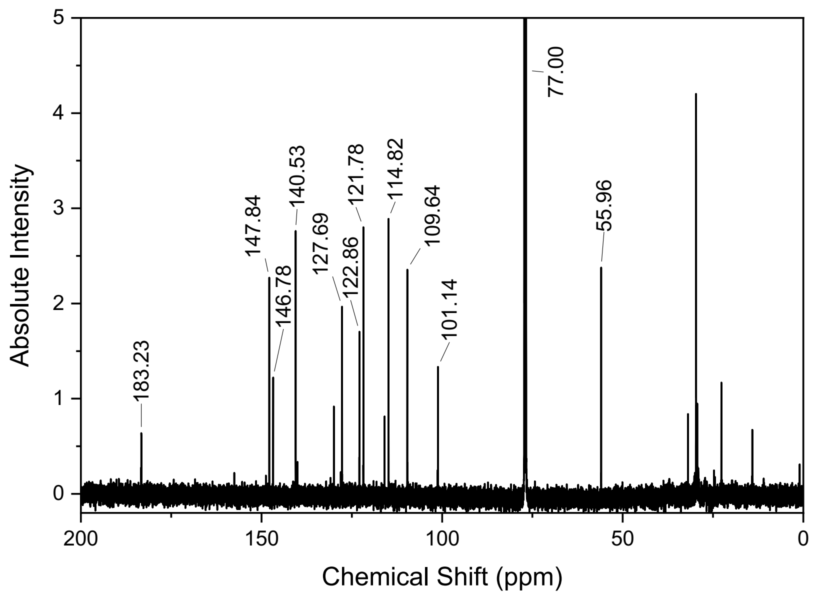

Figure S4. ^{13}C NMR spectrum of purified curcumin in $CDCl_3$.

Figure S5 shows the identification of C and H atoms in curcumin molecule.

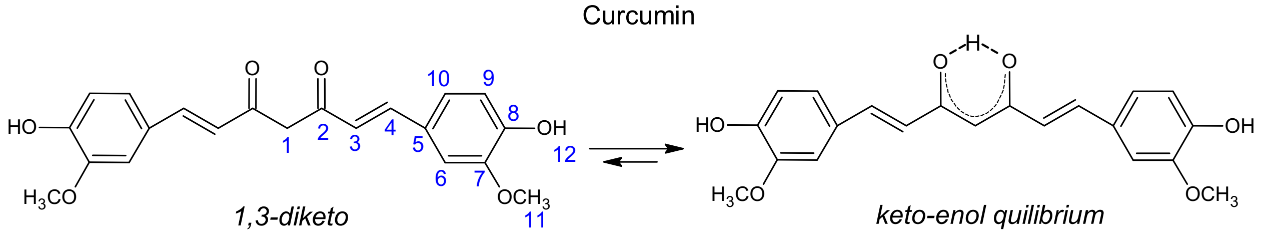

Figure S5. Identification of H and C atoms in curcumin molecule used in this work.

The results of our NMR analyses are summarized in Table S1 considering carbon/hydrogen numbering in curcumin molecule, which was used to assign the peaks in the NMR spectra (Fig. S5).

Table S1. ^{1}H and ^{13}C NMR chemical shifts of protons and carbons in extracted (EC) and purified curcumin (PC). In red are data from reference [1], in black of EC and in blue of PC.

H/C position (Fig. 5)	EC chemical shift (δ_H, ppm) in $CDCl_3$	PC chemical shift (δ_H, ppm) in $CDCl_3$	Multiplicity pattern	EC chemical shift (δ_C, ppm) in $CDCl_3$	PC chemical shift (δ_C, ppm) in $CDCl_3$
1	5.80	5.81)	s	101.16	101.14
2,2’	No H	No H		183.26	183.26
3,3’	6.46-6.50	6.47-6.51	d	121.80	121.78
4,4’	7.57-7.61	7.59-7.62	d	140.56	140.53
5,5’	No H	No H		127.71	127.69
6,6’	7.06	7.07	d	109.68	109.64
7,7’	No H	No H	d	147.88	147.84
8,8’	No H	No H	s	146.81	146.78
9,9’	6.94	6.95	d	114.86	114.82
10,10’	7.11	7.13	s	122.89	122.86
11,11’	3.95	3.96	s	55.9	55.96
12,12’	Not found	Not found		Not found	Not found
$CHCl_3$	7.26	7.27			
$CDCl_3$				77.07	77.00

The Heteronuclear Single Quantum Coherence (HSQC) analysis (Fig. S6), which correlates proton-carbon single bond is shown in Figure S6. As can be seen in this figure proton-carbon bonds match very well.

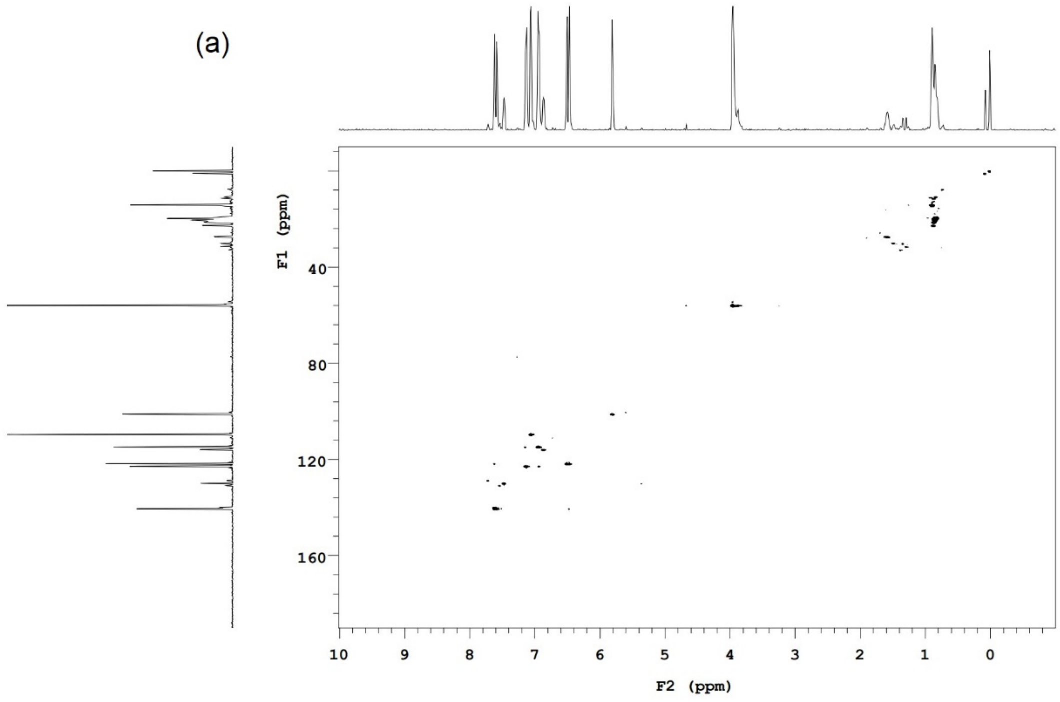

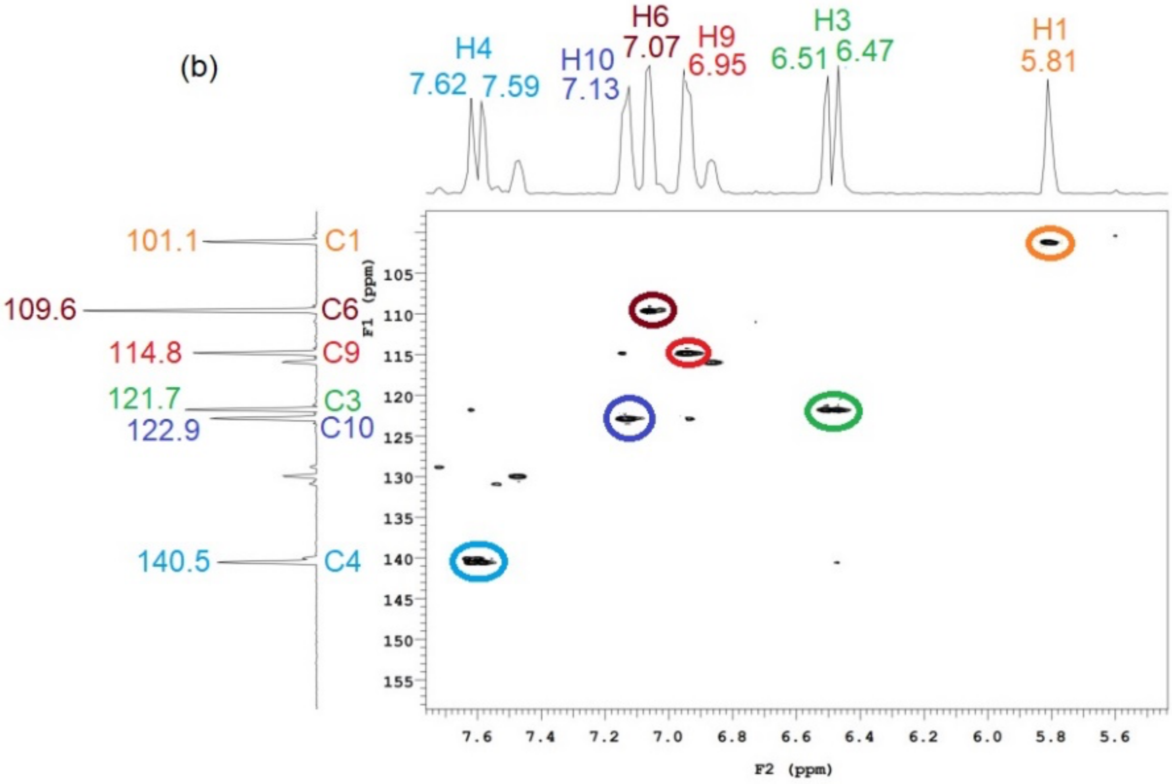

Figure S6. HSQC whole (a) and amplified (b) spectra of purified curcumin.

UV-Vis

For the UV-VIS analyses 40 g of commercial turmeric powder was dissolved in 100 mL of methylene chloride under reflux and magnetically stirred for 1 h at 38°C, which is this solvent's boil temperature at our altitude in Brazil. After that, the solution was filtered and the solvent was evaporated using a roto evaporator. The curcumin was precipitated with 40 mL of hexane, filtered again, and dissolved in CH_2Cl_2, C_2H_5OH, DMSO, NaOH 5 mol/L, and $CHCl_3$ for UV-Vis analyses. The UV-Vis spectra were recorded with a Jasco V-670 spectrophotometer.

Similar to the results shown in the main document it is clearly seen that curcumin is sensitive to the pH and relative polarity of the solvent, which was already seen by others. The maximum absorptions were 461, 429, 427, 416, and 415 nm for NaOH 5 mol/L, DMSO, C_2H_5OH, $CHCl_3$, and CH_2Cl_2 (Fig. S7), which are comparable to 469.3, 433.5, and 427.3 nm for solutions of curcumin in NaOH 5 mol/L, DMSO, and ethanol, respectively (Fig. 4 in the main text). Besides the main bands, there are also seen small bands due to the impurities as this curcumin was only extracted from turmeric powder and not purified.

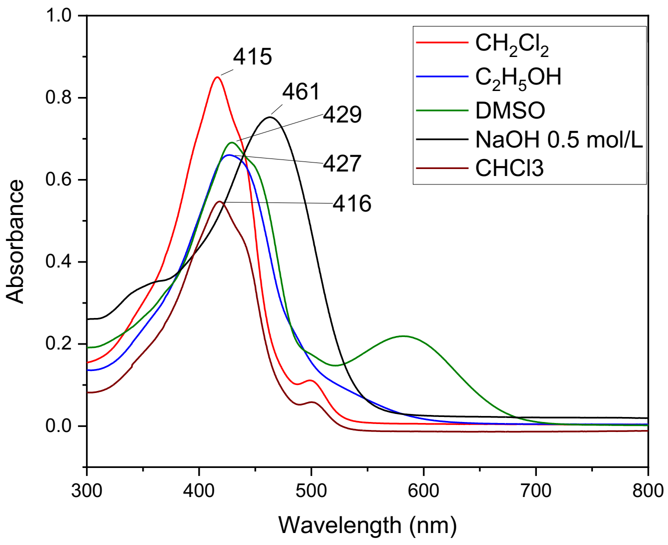

Figure S7. The UV-Vis spectra of curcumin solutions in CH_2Cl_2, C_2H_5OH, DMSO, NaOH 0.5 mol/L, and $CHCl_3$.

Table S2 summarizes physical chemical properties of CH_2Cl_2, C_2H_5OH, DMSO, NaOH, and $CHCl_3$ solvents used to dissolve curcumin and analyze its solutions by UV-Vis, shown in Figure S7.

Table S2. Summary of CH_2Cl_2, C_2H_5OH, DMSO, NaOH, and $CHCl_3$ relative polarities, polarity indexes, type of solvent, pH and curcumin peaks in these solvents.

**Solvent**	**Relative Polarity**	**Polarity Index**	**Type of Solvent**	**pH**	**Curcumin UV-Vis peak (nm)**
C_2H_5OH	0.654	4.3	Protic polar	7.33	427
DMSO	0.444	7.2	Aprotic polar	6-8	429
CH_2Cl_2	0.309	3.1	Aprotic polar		415
$CHCl_3$	0.259	4.1	Nonpolar	5-7	416
NaOH 5 mol/L			Polar	basic	461-469

FTIR results

The FTIR spectra of curcumin extracted from commercial turmeric powder (Fig. S8) and purified (Fig. S9) were obtained with IRAffinity-1 Shimadzu and KBr pellet.

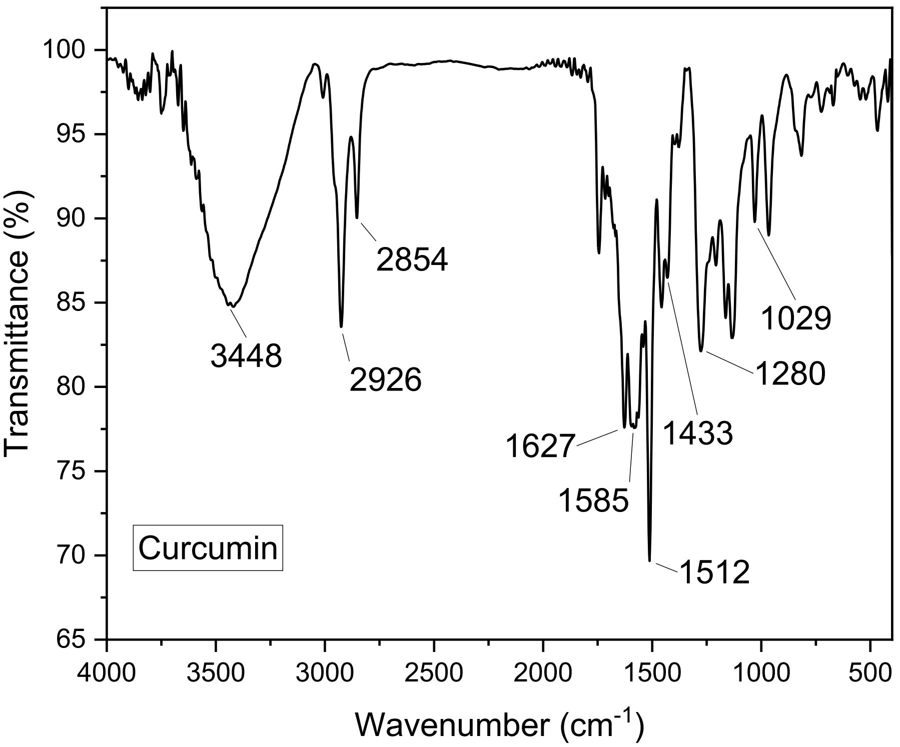

Figure S8. FTIR spectrum of extracted curcumin.

The characteristic FTIR bands of curcumin, seen in Figure S8, are very similar to those presented by Chen et al.[2]. OH stretching of phenolic rings peaks at 3448 cm^{-1} [2, 3]. At 2926 and 2854 cm^{-1} there are of aromatic and olefinic CH stretching vibrations and at 1433 cm^{-1} C-C vibrations; at 1627 cm^{-1} there is C=O stretching and at 1585 cm^{-1} there are C=C stretching and benzene ring vibrations [2, 3]. At 1512 cm^{-1} there are bending vibrations of C-H bound to benzene [3], C-O aromatic stretching vibrations appear at 1280 cm^{-1}, and C-O-C stretching vibrations are at 1029 cm^{-1}. The FTIR spectrum of purified curcumin (Fig. S9) has the same bands as extracted curcumin. The only difference is that bands at 2926 and 2854 cm^{-1} of the aromatic and olefinic CH stretching modes have higher transmitance in relation to the OH stretching of phenolic rings at 3423 cm^{-1}.

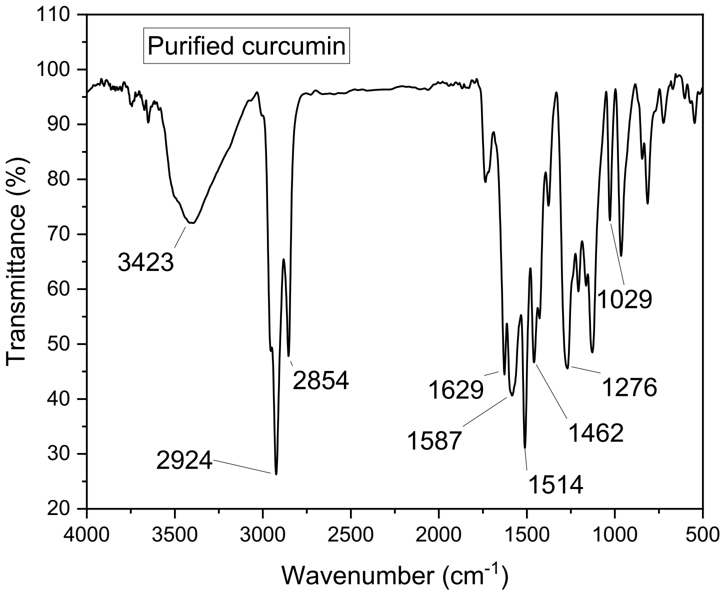

Figure S9. FTIR spectrum of purified curcumin.

References

1. Prasad, D.; Praveen, A.; Mahapatra, S.; Mogurampelly, S.; Chaudhari, S. R., Existence of β-diketone form of curcuminoids revealed by NMR spectroscopy. *Food Chem* **2021,** 360, 130000, doi: 10.1016/j.foodchem.2021.130000.
2. Chen, X.; Zou, L.-Q.; Niu, J.; Liu, W.; Peng, S.-F.; Liu, C.-M., The stability, sustained release and cellular antioxidant activity of curcumin nanoliposomes. *Molecules* **2015,** 20, (8), 14293-14311, doi: 10.3390/molecules200814293.
3. Gunathilake, T. M. S. U.; Ching, Y. C.; Uyama, H.; Hai, N. D.; Chuah, C. H., Enhanced curcumin loaded nanocellulose: a possible inhalable nanotherapeutic to treat COVID-19. *Cellulose* **2022,** 29, (3), 1821-1840, doi: 10.1007/s10570-021-04391-8.
